# A Persian validation of the burnout assessment tool

**DOI:** 10.1186/s12889-024-19314-y

**Published:** 2024-07-11

**Authors:** Simindokht Kalani, Mahla Dashti Esfahani, Payam Khanlari

**Affiliations:** 1 Department of Psychology, Faculty of Education and Psychology, University of Isfahan, Isfahan, Iran; 2 Department of Counselling, Faculty of Psychology and Education, Kharazmi University, Tehran, Iran; 3 Department of Occupational Health Engineering, School of Public Health, Tehran University of Medical Sciences, Tehran, Iran

**Keywords:** Burnout, Burnout assessment tool, Psychometric properties, Validation

## Abstract

**Background:**

Burnout is an increasing public health concern. Its prevalence has extended across diverse professions globally, posing significant challenges to individuals, organizations, and society. This phenomenon has undermined employee well-being, productivity, and organizational effectiveness, making it a critical concern in contemporary work environments. The present study aimed to examine the adaptation and assess the validity of the Persian version of the Burnout Assessment Tool (BAT).

**Methods:**

The adaptation process included the translation and back-translation of the BAT. Data were collected on a sample of 580 teachers using the convenience sampling. The BAT-Persian and Utrecht Work Engagement Scale were administered to collect the data. The reliability, factorial structure of the BAT-C and BAT-S, and the convergent and discriminant validity of BAT-C and work engagement were explored.

**Results:**

Confirmatory factor analysis supported a four-factor structure for the core dimensions (BAT-C; exhaustion, mental distance, emotional impairment, cognitive impairment), and a two-factor structure for the secondary dimensions (BAT-S; psychological distress, psychosomatic complaints). In the second-order model, the item loadings on the four factors of BAT-C ranged from 0.35 to 0.85, and on two factors of BAT-S ranged from 0.63 to 0.89. The Persian versions of the BAT-C and BAT-S showed good internal consistency (respectively, α = 0.95 and 0.90). Additional evidence supports the convergent and discriminant validity of the BAT-GR. the BAT‐C and its scales were negatively correlated with work engagement and dimensions (i.e., vigor, dedication, and absorption). Moreover, the BAT‐S and its scales negatively correlated with work engagement and dimensions.

**Conclusions:**

This study provided evidence that the Iranian version of BAT represents a reliable and valid tool for measuring burnout in the work context. A reliable and valid tool for assessing burnout in the Iranian workplace enables early detection of employee distress, allowing for timely intervention and support. This means that identifying the signs and symptoms of burnout in the early stages can prevent more severe consequences such as absenteeism, reduced productivity, or turnover.

**Supplementary Information:**

The online version contains supplementary material available at 10.1186/s12889-024-19314-y.

## Background

Teachers are a main component of the education system of any country. According to the Iranian Statistics Center, approximately 1.5 million individuals are actively engaged in the education sector in Iran [1], and teachers have a special place in Iranian culture. Due to factors like job nature, insufficient salaries and benefits, and conflicts with students and related organizations, teachers face significant work-related psychological pressures affecting their mental and physical health [2, 3]. One consequence of enduring mental pressure and chronic work stress is burnout. Burnout is prevalent in today’s fast-paced and demanding work environments, affecting individuals across various professions and industries [4]. It has been estimated that a wide range of US teachers (between 5 and 20%) exhibit burnout [5]. Some studies have reported the prevalence of burnout among Iranian teachers. In one study 3.3% and 5.2% of teachers had emotional exhaustion (EE) scores of 26–29 and > 30, respectively. In terms of depersonalization (DP), 29.1%, 62.1%, and 8.8% obtained < 6, 7–14, and > 15 scores on MBI, respectively. also, 97.3% and 2.7% of teachers had a personal accomplishment (PA) score of < 36 and 37–43, respectively [6]. In another study, 24% of participants reported high levels of burnout [7]. The World Health Organization includes the following definition of burnout in its International Classification of Diseases-11 document: “Burnout is a syndrome resulting from chronic workplace stress that has not been successfully managed. Burnout has been conceptualized as a combination of inability and unwillingness to expend the necessary effort at work to complete work properly. In this context, inability and unwillingness are two inseparable components [8, 9]. It is also characterized by exhaustion, cynicism, and reduced professional efficacy, which can harm the individual’s well-being and performance at work [10, 11]. Recognizing the importance of addressing burnout and its impact, researchers and practitioners have developed various tools and assessments to measure and evaluate burnout levels. There are many tools for measuring and evaluating burnout. Maslach Burnout Inventory (MBI) [12], Copenhagen Burnout Inventory [13], Burnout clinical subtypes questionnaire [14], and Shirom–melamed burnout questionnaire [15] are the most common Burnout measurement tools [16]. MBI has been the most widely used and reliable tool to measure burnout, but it also has weaknesses [16–18]. The first concern about burnout assessment by MBI is that the syndrome is undiagnosable (Bianchi et al., 2021). The practical applicability of the MBI for individual burnout assessment is poor. The main issue is that the MBI does not produce a single burnout score that can distinguish between burned-out and non-burned-out cases. The MBI manual explicitly states that each respondent’s scale scores should be calculated and interpreted separately, and responses should not be combined into a single “burnout” score. For practitioners to diagnose burnout effectively, a self-report questionnaire is essential. However, the MBI cannot fulfill this role as it was developed as a multi-dimensional research instrument rather than an individual assessment tool [18]. Second, the relationship between the burnout construct and its measurement by the MBI is unclear. The MBI defines burnout as a combination of exhaustion, cynicism, and inefficacy, but recommends assessing these components separately. This contradicts the formal definition of burnout and results in the MBI measuring three separate entities instead of burnout as a unified construct. On a related note, if exhaustion, cynicism, and inefficacy do not reflect a unified phenomenon, the reason for considering these three entities “the essential elements of burnout” ( [19], p. 1) is incomprehensible [20]. Third, measures of burnout often overlook key signs of job-related distress, such as work-related suicidal thoughts, which are significant predictors of suicide [18, 20]. This narrow focus has been criticized for decades. Additionally, burnout is linked to cognitive deficits [18, 21] and symptoms like irritability, sleep problems, and tension headaches, leading some to view burnout as a work-related form of neurasthenia [18, 22]. There is also an ongoing debate about whether burnout is a distinct condition or a form of depression, with evidence showing that symptoms of burnout and depression frequently co-occur [20, 23]. Moreover, the inclusion of reduced professional efficacy as a core aspect of burnout is questioned [24].Fourth, despite the MBI’s role in legitimizing the burnout construct and establishing the concept of “burnout syndrome,” its foundations are weak. The MBI was developed through rudimentary exploratory studies, disconnected from existing stress-related literature, and prone to researcher bias [20]. The creation of the MBI’s items involved unclear and arbitrary decisions, leading to skepticism about the scientific validity of the burnout construct (see [25] p.188). Additionally, the MBI has technical and psychometric issues, including extreme item wording and the artifact introduced by reversing positively worded items. Its Accomplishment and Depersonalization subscales show low reliability for important decisions like diagnosing burnout [26]. Additionally, the factorial validity is questionable, with studies suggesting emotional exhaustion and cynicism form a common factor, while professional efficacy is separate [20].

Considering these weaknesses of MBI, Schaufeli et al. [18] created a new burnout assessment tool (BAT). BAT is a new self-report questionnaire to measure burnout. The BAT consists of 33 items and measures the core dimensions (BAT-C) of burnout as well as the secondary dimensions (BAT-S). The BAT-C assesses the four core dimensions: (1) exhaustion (severe loss of energy, both physical as well as mental), (2) cognitive impairment (reduced functional capacity to adequately regulate one’s emotional processes), (3) emotional impairment (reduced functional capacity to adequately regulate one’s cognitive processes), and (4) mental distance (mental withdrawal and psychological detachment from the job). In addition, the BAT includes a secondary symptom scale with two factors: psychological complaints (e.g., sleep problems, tension, and worry) and psychosomatic complaints (e.g., headache, chest and muscle pain) [16, 18]. BAT considers burnout a second-order factor that functions as a syndrome, meaning that all four components are interconnected and belong to a higher-order construct, burnout [18, 27]. The invariance of the BAT measure is tested across seven samples of offending countries and BAT is shown to be consistent across countries for meaningful comparisons of burnout scores [28]. BAT has been translated and validated in several languages so far [17, 27, 29, 30] However, there is a lack of validated burnout assessment tools specifically designed for Persian-speaking populations. This gap in the literature highlights the need for a culturally sensitive and linguistically appropriate burnout assessment tool that can accurately measure burnout levels in Persian-speaking individuals. Cross-cultural validation of burnout assessment tools is essential for ensuring that individuals from diverse cultural and linguistic backgrounds are accurately identified and supported in managing burnout [31]. Consequently, this study aimed to validate the Persian version of BAT among a sample of Iranian teachers.

From the past until now, researchers in Iran have used the Maslach Burnout Inventory (MBI) for studying job burnout. However, there is no tool available that can accurately determine job burnout without the shortcomings of the MBI. Additionally, systematic reviews published in Iran, which investigate the prevalence of burnout, have not been able to establish a consistent cut-off point used by different studies to distinguish between burnout and non-burnout individuals (See [32–34](. Overall, the Persian validation of the Burnout Assessment Tool (BAT) represents an important contribution to the field of burnout research and practice. By providing a culturally sensitive and linguistically appropriate tool for measuring burnout levels in Persian-speaking populations, this validation study offers a valuable resource for researchers, practitioners, and organizations seeking to address burnout and promote well-being in the workplace.

## Methods

### Participants

We collected 580 responses through research questionnaires utilizing non-probability sampling. The average age of the respondents was 38.86 years (with a standard deviation of 9.30), spanning from 19 to 65 years old. Table 1 provides a summary of the demographic data.

**Table 1:** Sample demographic information

	n	%
Gender		
Male	496	85.5
Female	84	14.5
Length of employment (years of teaching experience)		
Less than 10 years	243	41.9
11–20	183	31.6
21–30	154	26.6
Education		
Diploma	6	1
Associate degree	13	2.2
Bachelor	336	57.9
Masters	211	36.4
Ph.D	14	2.4
Type of school		
Private School	113	19.5
Magnet schools	10	1.7
State school	436	75.2
Gifted school	21	3.6
Employment status		
Official	333	57.4
Contractual	247	42.6

*N* = 580

### Procedure

#### Data collection

This research received ethical approval from the Research Ethics Committees of the University of Isfahan (IR.UI.REC.1402.071). It employed a cross-sectional survey design, gathering data online through a questionnaire hosted on www.porsline.ir. The questionnaire link was disseminated through teachers’ social networks across 29 provinces of Iran from October 4, 2023, to February 4, 2024. Participation was voluntary, and confidentiality was assured for all respondents. Before participation, individuals provided informed consent and were informed of their right to withdraw at any point. To collect maximum data, we re-uploaded the questionnaire link in teachers’ social network groups at the beginning and middle of each day. In addition, as an incentive, we have announced that if someone wants to know about the burnout score and other variables under investigation, they can register an email address. Also, in some cases, we asked our friends to remind their teacher colleagues about completing the questionnaires. 608 participants completed the research scales, with 28 incomplete or ineligible questionnaires excluded from the analysis, yielding a response rate of 95.39%. We used the following criteria to determine the inclusion or exclusion of incomplete or ineligible questionnaires from the analysis:

- Questionnaires with a high proportion of missing responses (e.g., more than 10% of items unanswered) were excluded to avoid bias and ensure data integrity. For questionnaires with minor missing data, statistical methods such as imputation were used if the missing data was random and did not exceed the threshold.
- Questionnaires with responses that were inconsistent or contradictory (e.g., answering “Strongly Agree” to both positively and negatively worded items that should logically be inversely related) were excluded.
- Responses that showed suspicious patterns, such as answering all questions with the same option (e.g., selecting “Neutral” for all items) or extremely rapid completion times that suggested lack of attention, were excluded. If multiple submissions from the same respondent were detected, only one (usually the most complete and earliest submission) was included, and duplicates were excluded.

Furthermore, For questionnaires with minor missing data, statistical methods such as imputation were used if the missing data was random and did not exceed the threshold. By applying these criteria, the integrity and reliability of the data were maintained, ensuring that the analysis was based on high-quality and relevant responses.

#### Translation and back-translation

To translate BAT-C and BAT-S from English to Persian (or Farsi) language, the researchers adhered to standard guidelines for the phases of the translation and back-translation process, as outlined by Sousa and Rojjanasrirat [35]. Also, we used the GESIS guideline [36] to document the translation, which is available in the Excel file (in the supplementary material).

To translate the Burnout Assessment Tool (BAT) from English to Persian, we followed a systematic approach involving the following steps:

- *Initial Translation:* Initially, two researchers, both with a minimum of 5 years of experience as academics or organizational psychologists, one of whom is the author of the current paper, and proficient in both Persian and English languages (with Persian as their mother tongue), reviewed the initial translation of the scale into Persian.
- *Translation Reconciliation:* The two independent translations were compared and reconciled to create a single version. The degree of agreement between them was calculated using the Kappa coefficient. Also, any discrepancies between the translations were resolved through discussion.
- *Back-Translation:* The reconciled Persian version was back-translated into English by two different translators unaware of the initial translation process, and again, the degree of agreement between their translations was assessed. The results indicated a high level of agreement between the raters in both the translation and back-translation phases, confirming the accuracy of the translation.
- *Comparison with the Original Version:* The back-translated version was compared with the original English version to check for accuracy and consistency in meaning. Any semantic differences were identified and corrected.
- *Final Approval:* The final Persian version was reviewed and approved by twenty teachers who were enlisted to complete the Persian version of the BAT scale. They were asked to report any doubts, questions, or misunderstandings regarding the clarity of instructions, response format, and sentence structure. The finalized Persianized questionnaire is provided in Tables 2 and 3.

**Table 2:** Persian version of Burnout Assessment Tool—Core Symptoms (BAT-C)

	items
	خستگی
1	At work, I feel mentally exhausted هنگام كار، احساس مي كنم از لحاظ ذهني به شدت خسته ام
2	Everything I do at work requires a great deal of effort هنگامی که به کار مشغولم برای انجام هر فعالیتی باید انرژی زیادی صرف کنم
3	After a day at work, I find it hard to recover my energy پس از يك روز كاري، بازيابي انرژي برايم دشوار است
4	At work, I feel physically exhausted هنگام کار، از لحاظ جسمی احساس خستگي زيادی می کنم
5	When I get up in the morning, I lack the energy to start a new day at work وقتي صبح از خواب بيدار مي شوم، انرژي كافي براي شروع يك روز تازه کاری را ندارم
6	I want to be active at work, but somehow I am unable to manage دلم مي خواهد سر كار فعال باشم اما به نوعي از عهده آن بر نمي آيم
7	When I exert myself at work, I quickly get tired وقتي همه نيرويم را سر كار صرف مي كنم، به سرعت خسته مي شوم
8	At the end of my working day, I feel mentally exhausted and drained در پايان یک روز كاري، از لحاظ ذهني به شدت احساس خستگي و احساس بیحالی دارم
	فاصله ذهنی
9	I struggle to find any enthusiasm for my work باید خیلی تلاش کنم تا شوق کار کردن را در خود برانگیزم
10	At work, I do not think much about what I am doing and I function on autopilot هنگام انجام كار، خيلي به كارم فكر نمي كنم و بیشتر به صورت ماشینی و خودکار کار می کنم
11	I feel a strong aversion towards my job احساس بیزاری شدیدی نسبت به كارم دارم
12	I feel indifferent about my job نسبت به انجام كارم بی تفاوت هستم
13	I’m cynical about what my work means to others نسبت به اينكه ديگران در مورد كار من چه ديدگاهي دارند، احساس بد بيني دارم
	آسیب دیدگی شناختی
14	At work, I have trouble staying focused هنگام کار، برای حفظ تمرکز مشکل دارم
15	At work I struggle to think clearly هنگام كار، انگار ذهنم قفل می شود
16	I’m forgetful and distracted at work هنگام کار حواسم پرت می شود و فراموشکار می شوم
17	When I’m working, I have trouble concentrating وقتي در حال كار هستم، نمی توانم دقت و تمرکز داشته باشم
18	I make mistakes in my work because I have my mind on other things در كارم اشتباه مي كنم چون ذهنم درگير چيزهاي ديگر است
	آسیب دیدگی هیجانی
19	At work, I feel unable to control my emotions سر كار، احساس مي كنم قادر به كنترل هیجان¬هایم نيستم
20	I do not recognize myself in the way I react emotionally at work هنگام کار واکنش های هیجانی ام آنچنان شدید است که انگار خودم نیستم
21	During my work I become irritable when things don’t go my way در جريان كار، وقتي كارها آنطور كه دلم مي خواهد پيش نمي رود، ناراحت يا غمگين مي شوم
22	I get upset or sad at work without knowing why سر كار ناراحت يا غمگين مي شوم بدون اينكه بدانم چرا
23	At work I may overreact unintentionally هنگام کار بدون اينكه بخواهم، بيش از حد نسبت به موضوعي واكنش نشان مي دهم

The full BAT-C form (including the instructions to respondents) is available in Persian in the electronic supplementary material, together with how-to score items of the BAT

**Table 3:** Persian version of Burnout Assessment Tool – Secondary Symptoms (BAT-S)

	Items
	شکایات روانشناختی
1	I have trouble falling or staying asleep به خواب رفتن يا ادامه خواب، برایم مشکل است
2	I tend to worry هميشه به نوعي نگراني دارم
3	feel tense and stressed احساس تنش و استرس دارم
4	I feel anxious and/or suffer from panic attacks احساس اضطراب مي كنم و/يا دچار ترس شديد مي شوم
5	Noise and crowds disturb me سرو و صدا و شلوغي باعث آزارم مي شود
	شکایات روانتنی
6	I suffer from palpitations or chest pain تپش قلب يا درد قفسه سينه دارم
7	I suffer from stomach and/or intestinal complaints ناراحتي معده ومشکلات گوارشی دارم
8	I suffer from headaches سردرد دارم
9	I suffer from muscle pain, for example in the neck, shoulder, or back دردهاي عضلاني دارم مخصوصاً در نواحی پشت، گردن و شانه
10	I often get sick اغلب از نظر جسمی حالم خوب نیست

The full BAT-S form (including the instructions to respondents) is available in Persian in the electronic supplementary material, together with how-to score items of the BAT

During the translation and adaptation process, several cultural and linguistic nuances were addressed, for example, certain terms and concepts that might be unclear or interpreted differently in Persian culture were translated in a way that preserved the original meaning and emotional tone. also, efforts were made to ensure that the linguistic style of the questionnaire was familiar and understandable to Persian-speaking respondents, without altering the core content of the questions. Any potentially sensitive content that could be perceived differently in Persian culture was carefully modified.

### Measures of interest

All measures were used in their adapted version to Iranian contexts.

#### Burnout

Burnout was measured using the Burnout Assessment Tool (BAT), developed by Schaufeli, De Witte and Desart [37]. The BAT-C, consisting of 23 items, measures four core symptoms of burnout: exhaustion (8 items, e.g., “After a day at work, I find it hard to recover my energy”), mental distance (5 items, e.g., “I feel a strong aversion toward my job”), cognitive impairment (5 items, e.g., “At work I struggle to think clearly”), and emotional impairment (5 items, e.g., “I do not recognize myself in the way I react emotionally at work”). Additionally, 10 items (BAT-S) assess secondary symptoms: psychological complaints (5 items, e.g., “I tend to worry”) and psychosomatic complaints (5 items, e.g., “I suffer from headaches”). All items were rated on a five-point Likert scale ranging from 1 (never) to 5 (always). Responses were summed and averaged for each subscale. The BAT was translated from English to Persian using a back-translation procedure (see Translation and Back-Translation section).

Schaufeli, Desart and De Witte [18] have investigated the construct validity of BAT using exploratory factor analysis and confirmatory factor analysis. they reported that the four-factor structure for the core dimensions is best represented by one general burnout factor. in addition, their results demonstrated that a two-factor structure was found for the secondary dimensions. In their study, the convergent validity and discriminant validity with other burnout measures—including the MBI and OLBI—were demonstrated, as well as discriminant validity with other well-being constructs, such as work engagement and workaholism. Also, they reported that overall, the internal consistencies of the BAT-C and its four subscales were well above 0.70, above 0.81 for the BAT-S, and 0.95 for the total BAT-C.

#### Work engagement

To evaluate work engagement, we utilized the Persian version of the 9-item Utrecht Work Engagement Scale (UWES-9) developed by Hajloo [38]. Work engagement encompasses three key components: vigor (3 items, e.g., “At my work, I feel bursting with energy”), dedication (3 items, e.g., “I am enthusiastic about my job”), and absorption (3 items, e.g., “I feel happy when I am working intensely”). Participants rated the items of the UWES-9 on a scale ranging from 0 (never) to 6 (always).

Hajloo [20] determined the psychometric properties of the short form of the Utrecht Work Engagement Scale (UWES-9) in a part of Iranian society. Their results showed that the three dimensions of energy, dedication, and absorption in the UWES-9 have internal consistency based on Cronbach’s alpha coefficient. To determine convergent and divergent validity, they used the Maslach Burnout Inventory (MBI) [12] and the Job Satisfaction Scale [39]. They reported that the content, convergent, and divergent validity of the UWES-9 are favorable. Their results from exploratory and confirmatory factor analyses showed that, in the Iranian version of the UWES-9, as in the original form, the three related but distinct dimensions of energy, dedication, and absorption are present.

### Strategy of analysis

#### Preliminary analysis

The authors initially conducted an item analysis using SPSS 27 [40] to examine the psychometric properties of the items, including mean, standard deviation, skewness, and kurtosis. They utilized these analyses to ensure the robustness of the data. In interpreting the values of skewness and kurtosis, the authors considered values of |1| (e.g., [40]), |2| (e.g., [41]), and |3| (e.g., [42]) as optimal. Additionally, they referenced the literature [43], which suggests a maximum acceptable cut-off of |7| for kurtosis in samples larger than 300 subjects [44].

#### Construct validity

To check construct validity, we checked factorial validity and the relationship between BAT and work engagement.

### Factorial validity

Following recommendations for measures development [42], the authors conducted Confirmatory Factor Analysis (CFA) to assess the factorial validity of the BAT. CFA is a psychometric assessment technique utilized when the factor structure of a measure has been previously evaluated, and researchers aim to test the number of factors, their relationships, and the loadings of indicators on a different sample [45]. The comparison between the actual covariance matrix and the reproduced covariance matrix of the a priori hypothesized structural model is used to evaluate the goodness of fit of the indices [44]. This analytical strategy was implemented as part of a multistage approach.

Following Schaufeli, Desart and De Witte [18], we used 8 models to assess BAT-C AND BAT-S fully. For the core symptoms of the BAT-C, three models were assessed. The first model (Model 1) was a one-factor model, where all items were loaded onto a single general burnout factor. The second model (Model 2) was a 4-factor correlated model, assuming four distinct yet correlated factors: exhaustion, mental distance, and impaired emotional and cognitive control. As burnout is conceptualized as a syndrome comprising a cluster of interrelated symptoms reflecting an underlying psychological condition, a second-order model was also tested (Model 3). This hierarchical model posited four distinct factors serving as indicators of one overarching factor representing the core of burnout. This higher-order factor is presumed to account for the correlation among the four factors [46].

For the secondary symptoms of the BAT-S, two models were evaluated. Similar to the BAT-C, a one-factor model (Model 4) and a correlated factor model (Model 5) were tested. Model 4 assumed that all items loaded onto one general factor, while Model 5 posited two distinct factors: psychological and psychosomatic complaints.

Next, three additional models combined the core and secondary dimensions were tested. Model 6 adopted a correlated factor approach, suggesting the presence of six distinct factors. Moreover, two hierarchical models were tested. Model 7 proposed that all six distinct factors were optimally represented by a single overarching, second-order factor (i.e., burnout). Conversely, Model 8 suggested that the four core factors were best represented by a first general factor (i.e., the core of burnout). In comparison, the remaining two factors were best captured by a second general factor (i.e., secondary symptoms). This latter model aligns with the conceptualization of burnout distinguishing between core and secondary dimensions.

To assess the goodness-of-fit of the models, four fit indices were utilized [47]: Chi-square (χ2), comparative fit index (CFI), Tucker-Lewis index (TLI), and the Root Mean Square Error of Approximation (RMSEA). A model is considered to fit the data well when the CFI and TLI values exceed 0.90, preferably reaching 0.95, and the RMSEA is less than or equal to 0.06 [48].

### Relation with external variables

Pearson’s correlation coefficient (r), Average Variance Explained (AVE), and squared latent correlations (R^2) were calculated to examine the presence of convergent and discriminant validity [49].

We used the AVE index to check convergent validity. Statistically, convergent validity is established when the Average Variance Extracted (AVE) is > 0.50 [50] and indicates evidence of internal consistency within the structure. Discriminant validity is established to ascertain the distinctiveness of the constructs in the study.

We chose the work engagement variable to examine the discriminant validity.

#### Reliability

### Internal consistency

Scale reliability for the overall BAT-33 and its subscale scores, as well as other measures, was assessed using Cronbach’s alpha coefficient. Interpretation of Cronbach’s alpha follows cutoff values proposed by George and Mallery [51]: excellent (α ≥ 0.9), good (α ≥ 0.8), acceptable (α ≥ 0.7), questionable (α ≥ 0.6), poor (α ≥ 0.5), and unacceptable (α < 0.5).

### Composite reliability

In addition to internal consistency, composite reliability was assessed for the BAT core and secondary symptoms scale. Composite reliability employs factor loadings rather than item covariances [52], providing more accurate estimates, particularly for non-congeneric items with varying factor loadings. Values ranging from 0.7 to 0.9 are generally considered satisfactory to good [53].

## Results‍

### Construct validity

To check construct validity, we checked factorial validity and the relationship between BAT and work engagement and its results are presented below.

#### Factorial Validity

For the core symptoms of the BAT-C, three models were assessed. The first model (Model 1) was a one-factor model, where all items were loaded onto a single general burnout factor. Considering that in the first model, CFI and TLI are less than the permissible value of 0.9 and RMSEA is also higher than the permissible value of 0.06, model 1 is not approved. This means that all items of BAT-C are not loaded on one factor and there are probably several factors in this questionnaire.

Model 2 was a 4-factor correlated model, positing four separate but interconnected factors: exhaustion, mental distance, and impaired emotional and cognitive control. As burnout is conceptualized as a syndrome comprising a cluster of interrelated symptoms reflecting an underlying psychological condition, a second-order model (Model 3) was examined. This hierarchical framework proposed four distinct factors as indicators of an overarching factor representing the core of burnout. For the core symptoms, Model 1 did not fit the data, whilst Models 2 and 3 showed a much better and similar fit to the data.

In addition, two models were assessed for the secondary symptoms of the BAT-S. Model 4 suggested that all items were associated with a single general factor, whereas Model 5 proposed the existence of two separate factors: psychological complaints and psychosomatic complaints. For the secondary symptoms, Model 4 had the worst fit to the data (CFI < 0.9, TLI < 0.9, RMESA > 0.06). Model 5—which included the psychological and psychosomatic complaints factor (distress)—had a much better fit, albeit not optimal since CFI (0.88), TLI (0.86) were slightly below 0.90 and RMSEA (0.09) exceeded 0.08.

Model 6 utilizes a correlated factor approach, indicating the presence of six separate factors. Model 7 suggested that all six distinct factors were optimally represented by a single overarching, second-order factor, namely, burnout. Lastly, Model 8 indicates that the four core factors were most accurately represented by a primary general factor, referred to (CFI > 0.9, TLI > 0.9, RMESA < 0.06). Hence, the theoretically assumed distinction between core and secondary dimensions seems psychometrically viable.

#### Relation with External Variables

To examine the convergent and discriminant validity of the BAT-C and BAT-S about work engagement, Pearson’s correlation coefficient, Average Variance Explained (AVE), and squared latent correlations R^2^ were analyzed. The findings, presented in Tables 4 and 5, revealed that the AVE surpassed the square correlation R^2^ for both the BAT-GR latent factors, thus providing additional evidence supporting the convergent and discriminant validity of the BAT-GR. The findings, presented in Table 5.

**Table 4:** Model fit indices for the different BAT models

	Model	χ^2^ (Chi-square)	S-B χ^2^	df	CFI	TLI	RMSEA [90% CI]		Δχ^2^	*p*
Core symptoms										
1	Unidimensional model	1912.014	1.3078	230	0.739	0.712	0.112 [0.108- 0.117]			
2	Correlated 4-factor model	787.648	1.2736	224	0.912	0.901	0.066 [0.061- 0.071]	2vs.1	1124.4	< 0.0001
3	Second-order model (4 first order, 1 s order)	798.921	1.2794	226	0.911	0.900	0.066 [0.061- 0.071]	3 vs. 1 3 vs. 2	1113.1 11.273	< 0.0001 0.0035
Secondary symptoms										
4	Unidimensional model	1153.819	1.4381	35	0.711	0.628	0.235 [0.223- 0.246]			
5	Correlated 2-factor model	231.515	1.3148	34	0.949	0.932	0.100 [0.088- 0.112]	5 vs. 4		< 0.0001
	Core & secondary symptoms									
6	Correlated 6-factor model	1369.227	1.2304	480	0.918	0.910	0.057 [0.053- 0.060]			
7	Second-order model (6 first-order, 1 s-order)	1478.032	1.2324	489	0.909	0.902	0.059 [0.056- 0.063]	7 vs. 6	108.81	< 0.0001
8	Second-order model (6 first-order, 2 s-order)	1436.712	1.2337	488	0.913	0.905	0.058 [0.054- 0.061]	8 vs 6	67.485 41.32	< 0.0001 < 0.0001

The factor-loading matrices of the models and all correlations between the latent variables are available upon request from the first author

*χ2* chi-square, *S-Bχ2* Satorra–Bentler scaling factor for chi-square, *df* degrees of freedom, *CFI* comparative fit index, *TLI* Tucker–Lewis index, *RMSEA* root mean square error of approximation, *Δχ2* difference in chi-square, *Δdf* difference in degrees of freedom, *p p*-value

**Table 5:** Average Variance Explained (AVE) and square latent correlations R^2^ for work engagement (UWES) and BAT

	AVE	R^2^				
		1	2	3	4	5
1. Core symptoms of burnout (BAT-C)	0.547	1.000				
2. Secondary Symptoms of burnout (BAT-S)	0.663	0.714	1.000			
3. Work engagement	0.653	0.384	0.199	0.330	1.000	

*AVE* Average Variance Extracted, *R2* squared correlations

As expected, the BAT‐C and its scales were negatively correlated with work engagement and its dimensions (i.e., vigor, dedication, and absorption). Moreover, the BAT‐S and its scales were negatively correlated with work engagement, and its dimensions (see Table 6).

**Table 6:** Pearson’s r coefficient of variables

	1	2	3	4	5	6	7	8	9	10	11	12
1. Core symptoms of burnout (BAT-C)	1											
2. exhaustion	0.86	1										
3. mental distance	0.68	0.45	1									
4. cognitive impairment	0.82	0.54	0.49	1								
5. emotional impairment	0.82	0.55	0.47	0.69	1							
6. Secondary Symptoms of burnout (BAT-S)	0.68	0.58	0.38	0.56	0.65	1						
7. psychological complaints	0.69	0.58	0.38	0.57	0.66	0.90	1					
8. psychosomatic complaints	0.55	0.48	0.31	0.44	0.52	0.90	0.64	1				
9. Work engagement	-0.57	-0.52	-0.35	-0.48	-0.43	-0.36	-0.40	-0.26	1			
10. vigor	-0.57	-0.54	-0.34	-0.46	-0.43	-0.38	-0.41	-0.28	0.95	1		
11. dedication	-0.54	-0.48	-0.38	-0.46	-0.42	-0.33	-0.36	-0.23	0.94	0.84	1	
12. absorption	-0.48	-0.42	-0.27	-0.44	-0.38	-0.31	-0.35	-0.22	0.92	0.83	0.81	1

*N* = 580. All coefficients are significant at the 0.001 level

### Reliability

#### Internal consistency

The internal consistencies of the BAT-C and its four subscales were well above 0.70. Specifically, Cronbach’s alpha ranged from 0.65 to 0.87 for the subscales (exhaustion: 0.86, mental distance: 0.45, cognitive impairment: 0.87, and emotional impairment: 0.85), and it was 0.95 for the total BAT-C. Additionally, for the BAT-S, Cronbach’s alpha was 086, for both psychological and psychosomatic complaints.

#### Composite reliability

Composite reliability scores for the core symptoms were between 0.65 and 0.87 (exhaustion: 0.87, mental distance: 0.55, cognitive impairment: 0.87, and emotional impairment: 0.85), and secondary symptoms indices were 0.87 and 0.88, all showing good internal consistency [53].

## Discussion

The present study contributes to the international validation of the new burnout assessment tool, the BAT [37], by examining its psychometric properties in a diverse convenience sample of Iranian employees. Burnout is a widespread issue in the workplace, exacerbated by various stressors, and poses a significant challenge to employees’ health and well-being [54]. Although severe burnout cases are relatively rare, international data indicate that a significant portion of the workforce experiences milder forms of this syndrome [55]. Therefore, there is a pressing need for reliable and current instruments to assess the key symptoms of this detrimental psychological condition across different languages and occupational settings. This study presents the first validation results of the BAT in a Persian language context.

This study aimed to investigate the validity, reliability, and measurement invariance of the BAT a new burnout instrument. Schaufeli, Desart and De Witte [18] argued that the second-order factor model, distinguishing between core and secondary symptoms, aligns better with the theoretical understanding of the burnout construct and supports the view of burnout as a syndrome [37]. The BAT instrument supports this perspective by assessing burnout as a syndrome characterized by core symptoms (exhaustion, mental distance, emotional impairment, and cognitive impairment) and secondary symptoms (psychological distress and psychosomatic complaints), which may be linked to depressed mood and other comorbidities. Consequently, BAT treats burnout as a second-order factor, where all four components are interconnected and part of the same overarching construct, burnout [28]. The BAT consists of 33 items and measures the core dimensions (BAT-C) of burnout as well as the secondary dimensions (BAT-S). The BAT-C offers two ways to score: either a comprehensive burnout score or four-dimension scores. Similarly, the BAT-S provides two scoring alternatives: a holistic Secondary symptoms score or a two-dimensional score [56]. Following [18], we used eight models to assess BAT-C and BAT-S fully.

Our findings demonstrate that the BAT-Persian exhibits strong psychometric properties in terms of structure, construct validity, and reliability of scale scores. Our analyses support both a correlated four-factor model and a second-order model comprising four lower-order factors that represent the core symptoms of burnout: exhaustion, cognitive impairment, emotional impairment, and mental distancing. This is consistent with the conceptualization of burnout as a syndrome within the BAT framework [56] and extends previous international efforts to validate this structure across different cultures( e.g., [27, 28]). It is noteworthy that not all previous studies have examined both factor model specifications. Some studies have focused on a higher-order model based on Schaufeli, De Witte and Desart [37] conceptualization of burnout. For instance, De Beer, Schaufeli [28] found that a higher-order core symptom factor structure is robust across seven countries. Studies testing various model specifications have found comparable goodness of fit indices for both a correlated four-factor model and a higher-order model (e.g., [18, 57]). Our findings are supported by studies conducted in various countries, including Ecuador using BAT-23 [30] and Italy using BAT-23 [17]. Cumulative evidence indicates that the dimensions of the Burnout Assessment Tool (BAT) are consistent across different regions in Asia, America, and Europe [58]. Therefore, the findings are consistent with those observed in previous validation research conducted in other countries.

Additionally, we empirically supported a two-factor structure for the secondary symptoms of burnout, comprising psychological and psychosomatic complaints. As expected, the secondary symptoms were positively correlated with the core symptoms of burnout. Although these correlations were moderate in strength (ranging from 0.31 to 0.66), we did not observe a major overlap with the core symptoms, which aligns with the original theorizing behind the BAT. It’s worth mentioning that Confirmatory factor analysis (CFA) and High correlation coefficients between psychological and psychosomatic complaints indicate a distinction between psychological distress and psychosomatic complaints cannot be made. The results of the exploratory factor analysis of Schaufeli, Desart and De Witte [18] also showed that such a distinction cannot be made. However, Schaufeli, Desart and De Witte [18] noted that such a distinction was previously made by Terluin, van Marwijk [59]. Additionally, the authors of Symptom Checklist 90-Revised (SCL-90R) [60] distinguish among nine distinct subtypes of distress, including sleep issues and somatization. Nevertheless, the SCL-90R acknowledges that these various types of distress are interconnected, and it allows for the computation of a composite total score, which reflects the individual’s overall level of distress. Therefore, as mentioned by Schaufeli, Desart and De Witte [18], we conclude that while various types of distress may be discernible, they are strongly correlated with one another, particularly within a representative sample of the working population, and collectively signify a general form of distress. Therefore, it appears appropriate to calculate a composite distress score.

Our results demonstrated that not only six distinct factors can be identified in the idol, but all six distinct factors are optimally represented by a single overarching, second-order factor, namely, burnout. Lastly, results indicated that the four core factors were most accurately represented by a primary general factor, referred to as the core of burnout. In contrast, the remaining two factors were better described by a secondary general factor, termed secondary symptoms. This latter model is in line with the conceptualization of burnout that distinguishes between core and secondary dimensions [18]. Considering the satisfactory fit and the findings from the latent correlations within the six-factor model, the second-order model, which distinguishes between the core and secondary dimensions, is favored on theoretical grounds. BAT-Persian measures both core and secondary burnout symptoms, that have already undergone validation in various European and non-European contexts [9, 17, 27, 30, 57, 58, 61–65]. Namely, (a) burnout is considered a syndrome—that is compatible with the idea of a second-order model; and (b) a distinction between core and secondary burnout symptoms corresponds with the definition of burnout by Schaufeli, Desart and De Witte [18]. This is corroborated by a recent study of the Burnout Assessment Tool (BAT) conducted with a representative sample of Japanese workers [57]. The study confirmed that both a second-order factor model comprising only the core symptoms and a second-order factor model including both the core and secondary symptoms demonstrated a good fit to the data. Furthermore, another recent study discovered that the aforementioned second-order factor model remained consistent across seven cross-national representative samples from Austria, Belgium, Finland, Germany, Ireland, Japan, and The Netherlands [28].

Moreover, our findings lend support to the discriminant validity of the construct. BAT-C and BAT-S scores were negatively correlated with work engagement and its dimensions. Strong negative correlations between burnout and work engagement are what is theoretically expected from these two constructs [66, 67] and are in line with other research evidence [27]. This means that, as measured with the BAT-Persian, burnout can be distinguished from other psychological states such as work engagement. This distinction is crucial not only from a practical standpoint but also for advancing further research on the topic. Recent studies on work-related well-being have increasingly focused on understanding the co-occurrence of burnout (or its specific dimensions) with work engagement [68, 69] as well as their inter-relationships (e.g., [70, 71]). Therefore, high-quality measurement tools are essential for addressing these questions effectively.

Each of the burnout core and secondary symptom subscales—except mental distance- also demonstrated good internal consistency, as evidenced by high-scale reliability coefficients. The empirical evidence presented in our study aligns with findings from other countries [30, 57, 72], suggesting that even within the Iranian context, the BAT may serve as a conceptually robust and empirically reliable tool for assessing burnout in work environments. In the present study, only mental distance was the first-order dimension that had the lowest α, and ω(α = 0.45, ω = 0.55), as did in the Italian version [38], Ecuadorian version [30], and Portuguese version [27]. However, samples from other countries showed that mental distance did not present the lowest internal consistency estimates of all first-order dimensions [58].

### Limitations and recommendations for future research

Our study is subject to several limitations that warrant caution in interpreting the results. One limitation is that the examination of the studied variables relied solely on self-report questionnaires, introducing the risk of common method variance [73].

First, the non-probabilistic convenience sample we obtained introduces some degree of selection bias. However, it’s important to note that probabilistic sampling, where all units in the population have known and positive probabilities of inclusion, is only feasible when there exists a complete and up-to-date list of the members of the population being investigated [74, 75]. In our case, such a comprehensive list was not available. Even with large samples, the representativeness of the samples cannot be assumed if the sampling method is not probabilistic.

The second limitation of this study is the gender composition of the sample. The majority of the participants in this research were male, which may have influenced the results and limited the generalizability of the findings. To increase the accuracy and generalizability of the results, it is recommended that future studies use a more balanced gender sample.

Third, the current correlational study adopts a cross-sectional design. Longitudinal designs hold the potential to enhance the validity evidence of the BAT. Specifically, they enable the testing of longitudinal measurement invariance, thereby allowing for the examination of the stability of BAT’s structure over time. Incorporating longitudinal designs can provide valuable insights into the stability and reliability of BAT measurements over extended periods.

Fourth, the current manuscript focused solely on two out of the five sources of validity evidence outlined by [76]. Specifically, validity evidence based on the relations to other variables was examined only from a correlational perspective, with one related construct being analyzed. Further investigation across additional sources of validity evidence could provide a more comprehensive understanding of the Burnout Assessment Tool’s validity.

Fifth, further research on the BAT’s scores’ relations to other variables should expand to other conceptually linked constructs such as fatigue.

Sixth, future studies should aim to analyze test-criterion relationships using predictive or concurrent designs. This approach would provide valuable insights into how BAT scores predict relevant outcomes or correlate with existing measures in real-time. Additionally, investigating other sources of validity evidence, such as validity evidence based on response processes, would further enhance our understanding of the psychometric properties and applicability of BAT in various contexts. These endeavors would contribute significantly to the comprehensive validation of BAT as a reliable tool for measuring burnout.

Seventh, to check the discriminative power of the BAT and the possibility of identifying groups and individuals with different risk levels for burnout (low, moderate, and high), future studies should also include burnout patients. The use of a single burnout score, as provided by the BAT, offers a practical means of distinguishing between healthy employees and those who may be at risk or experiencing early symptoms of severe burnout. By incorporating burnout patients, researchers can better understand how well the BAT performs in differentiating between various levels of burnout severity, thus enhancing its clinical utility and applicability in both preventive and intervention contexts. Establishing clinically validated cut-off scores for burnout risk among Iranian employees is crucial. As this is currently lacking not only in Iran but also in many other countries [18, 77], future studies should consider combining self-reported data from the Burnout Assessment Tool (BAT) with medical interviews. This combined approach can help define specific cut-off scores for accurately identifying different levels of burnout risk among Iranian employees.

### Practical implication

Such findings have both research and practical implications. Our study contributes to the cross-cultural burnout literature by demonstrating that the construct is perceived similarly (i.e., it retains the same structure) in a linguistic context quite different from English. Translating measures into other languages is a significant challenge in psychological research and assessment practice, as linguistic differences in item interpretation can lead to measurement non-invariance [78]. The present findings indicate that, in a configurable sense, the BAT-Persian operates similarly to its original version, allowing for clear identification of the core dimensions of burnout. From a research perspective, this enhances cross-cultural comparability and replicability of findings, which is crucial given the global research efforts devoted to studying this phenomenon.

In line with international results on the BAT [28, 30, 57, 72] (e.g., references [48–51]), this study demonstrated that the BAT is a reliable, valid, and free-to-use tool in the Iranian context. Having a free-to-use instrument is crucial as it enables researchers to compare data across different countries, sectors, and professional roles.

In addition, a sound burnout measure that provides an overall burnout score, such as the BAT, is particularly relevant for psychosocial risk assessment and work-related organizational interventions. This tool can be used as a potential outcome of work-related stress risk assessments to identify the impact of organizational factors and work characteristics on workers’ well-being. Specifically, a single burnout total score is very helpful in developing cut-off scores that can assess burnout prevalence within groups, organizations, and countries. Furthermore, developing cut-off points is crucial for identifying employees at risk for burnout, enabling targeted preventive measures, and evaluating the effectiveness of interventions for burned-out employees.

## Conclusion

Our study offered preliminary and encouraging evidence regarding the psychometric properties of the Iranian adaptation of a recently developed instrument for assessing burnout, namely the Burnout Assessment Tool. Our study suggests that the Persian version of the BAT can serve as a viable alternative measure for assessing burnout. It encompasses the evaluation of the burnout syndrome as a whole (total score), as well as its core components and secondary symptoms. This feature allows for having a comprehensive score of the syndrome, which could be of particular importance for practical purposes, such as assessing burnout, and planning, and evaluating burnout interventions.

### Supplementary Information

Supplementary Material 1.

## Data Availability

The data presented in this study are available on request from the corresponding author.

## Abbreviations

BAT: Burnout Assessment Tool
BAT-C: Burnout Assessment Tool-Core
BAT-S: Burnout Assessment Tool- Secondary
MBI: Maslach Burnout Inventory
UWES-9: 9-Item Utrecht Work Engagement Scale
CFA: Confirmatory Factor Analysis
χ2: Chi-square
CFI: Comparative Fit Index
TLI: Tucker-Lewis Index
RMSEA: Root Mean Square Error of Approximation
AVE: Average Variance Explained
